# Analysis of 46 Cases of Spontaneous Perirenal Hemorrhage: A Retrospective Observational Study

**DOI:** 10.3390/jcm14092986

**Published:** 2025-04-25

**Authors:** Seon Beom Jo, Sun Tae Ahn, Mi Mi Oh, Sung Joon Park, Young-Hoon Yoon, Jong Wook Kim, Jung-Youn Kim

**Affiliations:** 1 Department of Urology, College of Medicine, Korea University, Seoul 08308, Republic of Koreamamah@hanmail.net (M.M.O.); 2 Department of Emergency Medicine, College of Medicine, Korea University, Seoul 08308, Republic of Korea; kuedpsj@hanmail.net (S.J.P.); yyh71346@naver.com (Y.-H.Y.)

**Keywords:** perirenal hemorrhage, Wunderlich syndrome, renal tumor

## Abstract

**Background**: This study investigated the clinical features, underlying causes, and management of patients with spontaneous perirenal hemorrhage (Wunderlich syndrome; WS). **Methods:** We retrospectively reviewed the records of patients hospitalized for WS at a single tertiary center between 2011 and 2024. All patients were evaluated for non-traumatic perirenal hemorrhage identified on computed tomography (CT) in the emergency department. Clinical variables, including age, underlying diseases, symptoms, hemodynamic instability, and hospitalization course, were analyzed. Laboratory test results, as well as radiological and pathological findings, were reviewed. **Results:** The study included 46 events from 38 patients, with a median (IQR) follow-up period of 32 (4–82) months. The most common presenting symptom was flank pain, observed in 44 cases (95.7%). Renal lesions, including visible tumors, were detected in 25 cases (54.3%), while 13 cases (28.3%) exhibited perirenal hematoma without a distinct lesion. Among seven patients with hemodynamic instability (systolic blood pressure < 90 mmHg), one underwent emergency embolization, and four required emergency surgical exploration. Surgical intervention was performed in 13 cases (28.3%), all involving nephrectomy, while radiologic embolization was attempted in seven cases (15.2%), with one patient later requiring delayed nephrectomy. The final diagnosis revealed renal cell carcinoma in eight cases (six patients), angiomyolipoma in 11 cases (six patients), renal cysts in six cases, acquired cystic kidney disease in six cases, sarcoma in three cases, perivascular epithelioid cell tumor in one case, lymphoma in one case, and chronic pyelonephritis in four cases; no specific disease was identified in six cases. During follow-up, six patients died; four of these deaths were directly related to WS or its underlying etiologies. **Conclusions:** WS is a potentially life-threatening condition, with benign or malignant renal masses being the most common causes. Although the advancement of interventional techniques has led to an increasing number of cases being conservatively managed, the possibility of renal malignancy should always be considered.

## 1. Introduction

Wunderlich syndrome (WS), first described by Théophile Bonet in 1679 and later classified by Carl Wunderlich in 1856, is a rare but potentially fatal condition characterized by spontaneous, atraumatic renal or perinephric hemorrhage. Typically, contrast-enhanced CT plays a crucial role in diagnosing WS by identifying the presence of a perirenal hematoma and possible renal lesions. In uncertain cases, MRI or contrast-enhanced ultrasound (CEUS) may provide further diagnostic clarity. WS is rare, with only limited comprehensive case series available to guide management. Therefore, accumulating robust clinical data is crucial for enhancing diagnostic accuracy and treatment strategies in this uncommon entity. This condition manifests acutely and is frequently associated with hypovolemic shock, posing significant diagnostic and therapeutic challenges [1,2]. The condition is much rarer than iatrogenic or traumatic renal hemorrhages, making its diagnosis and management more challenging [3,4].

The classical clinical presentation of WS was described by Lenk’s triad, which included sudden-onset flank pain, a palpable flank mass, and hypovolemic shock [1,5]. However, this triad is observed in approximately 20% of cases, while most patients present with isolated flank pain, nausea, vomiting, or hematuria [4].

Several years ago, the authors published a study analyzing WS [4]. To date, most studies on WS are case reports or small-scale retrospective studies involving a small number of patients [3,6,7,8,9,10]. Furthermore, systematic reviews on WS are scarce and often outdated. Zhang et al. [5] highlighted the need for more comprehensive investigations into the etiology, clinical presentation, and management of this syndrome. Given the rarity of WS and the limitations of existing studies, we aimed to further investigate this condition by analyzing additional cases.

This study aimed to evaluate the clinical characteristics, underlying etiologies, and treatment outcomes of patients with WS, with the goal of providing valuable insights and contributing to the development of standardized management strategies.

## 2. Materials and Methods

### 2.1. Data Collection

We conducted a retrospective chart review of clinical events of WS at a single tertiary center between 2011 and 2024. We identified all patients who were hospitalized with a confirmed diagnosis of WS during this interval, ensuring consistent data collection and thorough record review throughout the study. Data on the following clinical variables were collected: age, sex, underlying medical conditions, presenting symptoms, presence of shock, hospitalization status, surgical intervention, and radiologic intervention. In patients who underwent surgical treatment, the pathological results from surgical specimens were considered the definitive diagnosis. For patients managed without surgery, the radiological interpretation from CT or magnetic resonance imaging (MRI) was considered the final diagnosis.

#### 2.1.1. Inclusion Criterion

All patients presented to the emergency department with flank pain or hematuria and were diagnosed with perirenal hemorrhage on computed tomography (CT) performed in the emergency setting.

#### 2.1.2. Exclusion Criterion

Patients with any history of trauma were excluded. 

### 2.2. Ethical Considerations

We received approval for this study from the Institutional Review Board of Korea University Guro Hospital (IRB No. 2025GR0168). The requirement for informed consent was waived by the IRB due to the retrospective nature of the study and the use of fully de-identified data. As no personal identifiers or sensitive information were included in the dataset, the IRB determined that the study posed minimal risk to participants and met the criteria for waiver of consent in accordance with the relevant ethical guidelines.

### 2.3. Data Analysis

All statistical analyses were performed using IBM SPSS Statistics 20 Network Version (Armonk, NY, USA) and Microsoft Excel (Microsoft Corporation, Redmond, WA, USA). Continuous variables (e.g., age, follow-up duration, and laboratory values) are expressed as median (IQR), and categorical variables (e.g., presence of flank pain and type of intervention) are presented as frequencies and percentages. We used Fisher’s exact test to compare categorical variables (e.g., the proportion of patients receiving surgery vs. embolization) between subgroups. A *p*-value < 0.05 was considered statistically significant.

## 3. Results

The study included 46 events from 38 patients, with a median (IQR) follow-up period of 32 (4–82) months. (See Table 1 for demographics.)

### 3.1. Patient Demographics and Medical History

Among the 38 patients, 23 were male and 15 were female. The median (IQR) age at the time of presentation was 39.5 (34–58) years.

Regarding underlying medical conditions, 14 patients had hypertension, eight had diabetes mellitus, 12 had chronic kidney disease (CKD), and 11 were undergoing dialysis at the time of presentation.

In addition, one patient had a history of cerebrovascular accident (CVA), two patients had atrial fibrillation (AF), and one patient had undergone aortic valve replacement (AVR). All four of these patients were taking some form of antiplatelet or anticoagulant therapy (aspirin, clopidogrel, apixaban, or warfarin).

### 3.2. Clinical Presentation

Flank pain was the most common symptom, observed in 44 out of 46 cases (95.7%). Other symptoms included fever (six cases, 13.0%), a palpable flank mass (five cases, 10.9%), and voiding difficulty or dysuria (five cases, 10.9%).

### 3.3. Hematuria and Urinalysis Findings

Gross hematuria was noted in three cases (6.5% of the total cohort, *n* = 46), while microscopic hematuria (defined as >5–9 red blood cells per high-power field) was found in 16 cases (34.8%). Due to dialysis dependence, urinalysis could not be performed in 13 cases (28.3%), involving 10 distinct patients.

### 3.4. Radiologic Findings

On CT imaging, renal lesions, including visible tumors, were identified in 25 cases (54.3%), while 13 cases (28.3%) showed only a perirenal hematoma without a distinct renal lesion. 

Treatment Outcomes

Among the 46 cases, 13 cases (28.3%) underwent surgical intervention, with all patients undergoing exploration and nephrectomy.

Radiologic intervention was performed in seven cases (15.2%), with one patient requiring delayed nephrectomy following initial embolization.

### 3.5. Hemodynamic Instability and Emergency Management

Shock (defined as systolic blood pressure < 90 mmHg) was present in seven cases (15.2%). Among these patients, one underwent emergency embolization, while four underwent emergency surgical exploration due to persistent hemodynamic instability.

### 3.6. Mortality and Follow-Up

During the follow-up period, six patients expired; four of these deaths were directly related to WS or its underlying etiologies.

### 3.7. Final Diagnosis

For patients who underwent surgical treatment, the pathologic diagnosis was considered definitive. In non-surgical cases, the radiologic interpretation (CT or MRI findings) was used as the final diagnosis.

Pathologic results were available for 17 cases, and the final diagnoses included the following (see Table 2).

Excluding duplicate cases, we analyzed 38 patients by dividing them into an early and a late period.

Early period: RCC was observed in three cases and AML in three cases, with 12 patients undergoing surgery, two receiving radiologic intervention (one of whom required surgery after intervention), and seven managed conservatively.

Late period: RCC was observed in two cases and AML in three cases, with only one patient undergoing surgery, six receiving interventional treatment, and 12 managed conservatively.

While there was little difference in the underlying causes between the two periods, there was a notable shift in treatment patterns.

In the first half, surgery was more commonly performed due to the limited availability of radiologic intervention. In the second half, 12 out of 19 (63.1%) WS cases were managed conservatively, while six out of 19 (31.6%) underwent radiologic intervention. Only one patient required surgery (nephrectomy). This difference was significant (*p* < 0.01).

## 4. Discussion

WS remains a rare and poorly understood clinical entity, largely due to its low incidence and the limited number of reported cases. Most of the existing literature consists of case reports and small-scale retrospective studies, with systematic reviews identifying only a handful of cases, further underscoring the rarity of this condition.

Historically, between 1985 and 1999, three meta-analyses reviewed a total of 165 cases reported in the English literature, highlighting the low incidence and under-recognition of WS [5]. A subsequent systematic review covering the period from 2000 to 2016 identified only 102 cases across various publications, reflecting the continued scarcity of comprehensive WS studies in modern clinical practice [3].

More recent single-center studies, such as those by Kim et al. [4] and Elbaset et al. [11], have analyzed 26 and 42 cases, respectively, demonstrating larger case series compared to previous reports. However, even with advanced imaging techniques such as CT and MRI, approximately 24% of cases in these studies had an undetermined source of hemorrhage, emphasizing the diagnostic challenges associated with WS [10,11].

The diversity of WS etiologies further complicates its diagnosis and management. While renal neoplasms, including AMLs and RCCs, account for most cases, vascular pathologies such as renal artery aneurysms, vasculitis syndromes (for example, polyarteritis nodosa), and renal vein thrombosis also contribute significantly [1,5,12].

Prompt diagnosis through contrast-enhanced CT is essential to identify the source of hemorrhage and prevent life-threatening complications. In cases where an underlying etiology remains unclear, MRI or angiography may be warranted for further evaluation [7,9,12,13,14].

The etiological spectrum of WS has evolved over time. Historically, RCC was the predominant cause of WS-related hemorrhage. However, over the past two decades, AML has surpassed RCC as the leading etiology, possibly due to the increased incidental detection of RCC through widespread imaging before the occurrence of hemorrhagic events. Currently, neoplasms account for approximately 57–63% of WS cases, with AML and RCC being the most frequently implicated tumors. Vascular causes, such as polyarteritis nodosa, renal artery aneurysms, arteriovenous malformations, and renal infarcts, contribute to 18–26% of cases, while infections account for 7–10%. Less common causes include cyst rupture, preeclampsia, and idiopathy [3,5,14,15,16,17].

Although our data do not show a clear statistical decline in RCC-caused WS, it is plausible that improved imaging and earlier detection of RCC may reduce the likelihood of patients presenting with hemorrhage [18]. Larger studies are needed to confirm whether RCC-induced WS is indeed decreasing over time. The proportions of RCC and AML remained consistent across different periods, while there was a slight increase in cystic disease associated with CKD.

Treatment strategies for WS are highly dependent on the patient’s hemodynamic stability. Hemodynamically stable patients can often be managed conservatively with fluid resuscitation, blood transfusions, and close monitoring, while those in hemodynamic shock require urgent intervention [1,19].

The management approach for WS has also shifted significantly in the last two decades. Before 2000, surgical intervention, including radical nephrectomy, was the primary treatment modality due to the lack of minimally invasive alternatives. However, advancements in interventional radiology have expanded the role of selective transarterial embolization (TAE) as a minimally invasive and effective first-line treatment for active bleeding, thereby reducing the need for nephrectomy in many cases [20]. A meta-analysis covering WS cases from 2000 to 2016 reported that 29.4% of patients were managed conservatively, 42.2% underwent TAE, and 27.5% required surgical intervention, reflecting a growing preference for organ-preserving treatments and the recognition that many cases of WS can be successfully managed without nephrectomy, particularly in hemodynamically stable patients [3,21,22,23].

In our study, treatment approaches evolved over time. This shift was not guided by a formal protocol but rather by each patient’s hemodynamic stability, the expanding availability of interventional radiology, and clinical discretion. In earlier years, surgery was frequently chosen due to limited IR resources, but as facilities improved, embolization or conservative approaches became feasible for stable patients.

For hemodynamically stable patients without strong suspicion of malignancy, a conservative approach with close monitoring can be effective. Meanwhile, selective transarterial embolization (TAE) offers a minimally invasive way to control active bleeding and potentially preserve renal parenchyma [20,21,22,23]. Surgery, including nephrectomy, remains indispensable for patients presenting with massive hemorrhage or suspected malignant lesions, especially when immediate oncologic control is paramount. This three-pronged strategy (conservative vs. TAE vs. surgery) aligns with the emerging literature that underscores an organ-preserving philosophy in stable WS cases, while ensuring prompt intervention for unstable presentations.

Moreover, while contrast-enhanced CT was the mainstay imaging modality during acute presentation, recent studies suggest that CEUS may provide additional characterization of renal masses, particularly in stable patients with potential AML [24]. CEUS can help delineate microvascular patterns and differentiate AML from RCC, reducing unnecessary invasive procedures [25]. Although we did not routinely perform CEUS in our series, it remains an area of interest for future diagnostic protocols, especially for patients who do not require immediate surgical intervention.

Despite providing one of the largest single-center case series on WS, this study has several limitations. First, it is a retrospective analysis in which selection bias and missing data are possible. Second, the study is confined to a single tertiary center, limiting the generalizability of our findings. Third, we did not implement a standardized diagnostic or treatment protocol, so therapeutic approaches (e.g., surgery, radiologic intervention, or conservative management) were chosen at the clinicians’ discretion. Fourth, the absence of a separate control group and the diversity of underlying renal pathologies make it difficult to generalize outcomes beyond this cohort. Finally, although we offer a relatively long median follow-up, we acknowledge that variations in follow-up intervals and loss to follow-up might constrain the reliability of our conclusions regarding long-term recurrence and mortality rates. Future multi-center, prospective studies with larger cohorts and extended follow-up periods are needed to validate these findings and refine treatment strategies for WS.

This study, despite being a single-center, retrospective analysis, provides one of the largest case series of WS reported to date, offering valuable insights into its etiology, diagnosis, management, and mortality. WS remains a challenging condition due to its diverse etiological spectrum and variable clinical presentation, emphasizing the need for larger, multi-center studies to establish more definitive diagnostic criteria, optimize management strategies, and improve patient outcomes.

## 5. Conclusions

In this single-center retrospective analysis of 46 spontaneous renal rupture events (Wunderlich syndrome), we found that angiomyolipoma and renal cell carcinoma were the most common underlying causes. Although advancements in interventional radiology have facilitated conservative or minimally invasive approaches, surgery remains indispensable for patients with hemodynamic instability or suspected malignancy. Further multicenter prospective studies are warranted to establish standardized guidelines and clarify long-term outcomes.

## Figures and Tables

**Table 1:** Clinical characteristics and laboratory test results of patients with Wunderlich syndrome.

	No. (Percentage)	Median (IQR)
Number of events	46	
Number of patients	38	
Age (y) at the time of presentation		39.5 (34–58)
Performed exploration or nephrectomy	13 (28.3%)	
Duration from admission to exploration (d)		10 (4.5–16)
Performed radiologic intervention	7 (15.2%)	
Follow-up duration (mo)		32 (4–82)
Gender		
Male	23 (53.8%)	
Female	15 (46.2%)	
Underlying diseases (per person)		
Hypertension	14 (36.8%)	
Diabetes	8 (21.1%)	
Chronic kidney disease	12 (31.6%)	
Dialysis	11 (23.1%)	
Tuberous sclerosis	1 (3.8%)	
Cerebrovascular accident (CVA)	1 (2.6%)	
Atrial fibrillation (AF)	2 (5.3%)	
Aortic valve replacement (AVR)	1 (2.6%)	
Symptoms and signs (per cases)		
Fever	6 (13.0%)	
Flank pain	44 (95.7%)	
Gross hematuria	3 (6.5%)	
Palpable flank mass	5 (10.9%)	
Voiding difficulty or dysuria	5 (10.9%)	
Microscopic hematuria (>5–9/HPF)	16 (34.8%)	
Urinalysis not performed	13 (28.3%)	
Shock (systolic blood pressure < 90 mmHg)	7 (15.2%)	
Laboratory data (per case)		
Hemoglobin (gm/dL)		10.9 (9–11.85)
WBC (×10^3^/µL, peak level)		11.00 (7.95–14.55)
ANC (×10^3^/µL, peak level)		9.03 (5.80–11.38)
Platelets (×10^3^)		214 (170.5–265.5)
PT (s)		13.75 (12.7–14.3)
aPTT (s)		35 (33.25–38.25)
CRP (mg/L, peak level)		5.32 (1.54–28.99)

HPF, high-power field; WBC, white blood cells; ANC, absolute neutrophil count; PT, prothrombin time; aPTT, activated partial thromboplastin time; CRP, C-reactive protein.

**Table 2:** Final diagnosis of patients with Wunderlich syndrome.

	No. of Cases (Percentage)
AML	11 (23.9%) (6 patients)
RCC	8 (17.4%) (6 patients)
Simple renal cyst	6 (13.0%)
ACKD	6 (13.0%)
Idiopathic	6 (13.0%)
Chronic pyelonephritis	4 (8.8%)
Sarcoma	3 (6.5%)
PEComa	1 (2.2%)
Lymphoma	1 (2.2%)

AML, angiomyolipoma; RCC, renal cell carcinoma; ACKD, acquired cystic kidney disease; PEComa, perivascular epithelioid cell tumor.

## Data Availability

The datasets generated and/or analyzed during the current study are available from the corresponding author upon reasonable request.
